# Exploiting Temporal Network Structures of Human Interaction to Effectively Immunize Populations

**DOI:** 10.1371/journal.pone.0036439

**Published:** 2012-05-07

**Authors:** Sungmin Lee, Luis E. C. Rocha, Fredrik Liljeros, Petter Holme

**Affiliations:** 1 IceLab, Department of Physics, Umeå University, Umeå, Sweden; 2 Department of Sociology, Stockholm University, Stockholm, Sweden; 3 Department of Energy Science, Sungkyunkwan University, Suwon, Korea; University of Zaragoza, Spain

## Abstract

Decreasing the number of people who must be vaccinated to immunize a community against an infectious disease could both save resources and decrease outbreak sizes. A key to reaching such a lower threshold of immunization is to find and vaccinate people who, through their behavior, are more likely than average to become infected and to spread the disease further. Fortunately, the very behavior that makes these people important to vaccinate can help us to localize them. Earlier studies have shown that one can use previous contacts to find people that are central in static contact networks. However, real contact patterns are not static. In this paper, we investigate if there is additional information in the temporal contact structure for vaccination protocols to exploit. We answer this affirmative by proposing two immunization methods that exploit temporal correlations and showing that these methods outperform a benchmark static-network protocol in four empirical contact datasets under various epidemic scenarios. Both methods rely only on obtainable, local information, and can be implemented in practice. For the datasets directly related to contact patterns of potential disease spreading (of sexually-transmitted and nosocomial infections respectively), the most efficient protocol is to sample people at random and vaccinate their latest contacts. The network datasets are temporal, which enables us to make more realistic evaluations than earlier studies—we use only information about the past for the purpose of vaccination, and about the future to simulate disease outbreaks. Using analytically tractable models, we identify two temporal structures that explain how the protocols earn their efficiency in the empirical data. This paper is a first step towards real vaccination protocols that exploit temporal-network structure—future work is needed both to characterize the structure of real contact sequences and to devise immunization methods that exploit these.

## Introduction

A key to effective prevention of infectious diseases is to identify people at risk. Such individuals can then be tested (especially for frequently asymptomatic diseases), informed of their risk situation (with the goal to change their risk behavior), or (if a vaccine exist) vaccinated. In this article, we will use vaccination, or immunization, as a metaphor for all these cases (but discuss some more concrete scenarios in the Discussion section).

Vaccination of an entire community is often not possible due to limited supply, production capacity and manpower. But to vaccinate a whole community is not desirable either—vaccine is expensive, it may have side effects and, luckily, it is not needed to immunize a community. If a large enough fraction *f* of it is vaccinated, a disease cannot spread to any substantial degree—the community has in effect achieved *herd immunity* [1]. Lowering the threshold of *f* to reach herd immunity is thus important and the way to do it is find people in risk of getting and spreading the disease and vaccinate them.

Epidemic outbreaks of an infectious disease are complex functions of both the characteristics of the pathogen and the movement and interaction patterns of the people [1]. The diversity in people's contact patterns carries over into disease spreading [1], [2]. It is believed that an outbreak such as the SARS epidemics of 2003 might not have become a major event if not for a few highly influential spreaders [3] exhibiting behavior far outside the norm. To lower the threshold for herd immunity, it is crucial to identify and vaccinate these potentially influential individuals. The idea in this paper is to use empirical contact structures, more or less close to those over which disease may spread, to identify important people to vaccinate. One early example of this approach is the *neighborhood vaccination* (*NV*) [4] protocol—choose a person at random among all persons that have been involved in at least one contact at time t*, ask her to name someone she met, vaccinate this other person, and repeat until a desired fraction of the vertices are vaccinated. Chances are high that this other person has a large degree (number of neighbors) in the static interaction network and may be influential in spreading disease. The contact structure thus not only influences disease dynamics, it is also a source of information that can be exploited to stop the disease. Human interaction patterns have much more structure that can be utilized in immunization protocols than merely the distribution of degrees in a static network, which is what neighborhood vaccination protocols build on. There is a great deal of temporal structure as well [2]. The simplest such patterns are cyclic—we are more likely to meet others at 3PM than at 3AM. Another potentially important temporal pattern is a broad distribution of contact rates between pairs of individuals [1]. Especially for diseases with a relatively high infectious dose, needing a prolonged exposure to transfer, this could have an impact on the disease dynamics that is hard to predict from network structure alone [5]. A straightforward extension of the *NV* protocol to capture this structure would be to ask the person chosen at random to name the person she has met most often since some specific time. This is one of the protocols we test. A third temporal pattern, which static network models do not capture, is the overturn of relationships, i.e. that an edge is active for a limited period of time and never again after this. If there is a positive correlation between the activity over an edge and the activity of the vertices at either side, then it is important to vaccinate people who are engaged in a period of activity. This leads to another extension of the *NV* protocol—ask the individual picked at random who her most recent contact was (who could spread the disease), and then vaccinate that person. Just like the *NV* protocol, this is a method does not require any global knowledge and can be implemented in practice.

To briefly review subsequent developments, following the *NV* method, one line of research has focused on exploiting higher-order static network structure [6]–[9]. This type of immunization protocol has the obvious disadvantage that higher-order structure is even more difficult to extract from social systems than the degree sequence of the contact network. This approach is perhaps best suited to stopping outbreaks of computer viruses where one can get a fuller picture of the transmission trees. Ref. [6] includes an iterated version of *NV* where neighbors of vaccinees, rather than neighbors of random individuals, are vaccinated. *Recent* and *Weight* can easily be extended to iterated versions (or to exploit higher order structure, like Refs. [7]–[9]). Another recent theme addresses the game theory aspect of voluntary vaccination [10]. If the majority of a community gets vaccinated, the community has herd immunity and there is no need to vaccinate an as yet unvaccinated person. On the other hand, if few people get vaccinated, the risk of getting the disease grows and vaccination may seem reasonable even to needle-phobics. The present work applies to scenarios of voluntary vaccination as well, provided that there is no strong correlation between an individual's contact-structural behavior and her willingness to get vaccinated if faced with an approaching epidemics. Yet other network-epidemiological studies of community immunization focus on the simultaneous effects of the population's response to the disease and that of a vaccination campaign [11], [12].

In the rest of the work, we will test the vaccination protocols mentioned above on four empirical datasets (some representing realistic contact structures for disease contagion, some representing other types of contact and included more from as a reference). Then we test the efficacy of the protocols by looking at how much the vaccination lower the upper bound of outbreak sizes (in fractions of the population size), and average outbreak sizes in Susceptible–Infected–Susceptible (SIS) simulations [1], averaged over the all individuals as infection sources. Throughout the paper, we compare our protocols to NV, both because it one of the best protocols that exploits only the contact structure and (more importantly) that our protocols reduce to NV if the temporal structure is projected out of the data. Then we go more into detail in explaining how the protocol performance relates to the temporal aspects of the contact structure. To this end, we use models generating temporal contact sequences with certain stylized features of the real data and study them by simulations and approximate analytical calculations.

## Results

### The protocols

**Figure 1:**
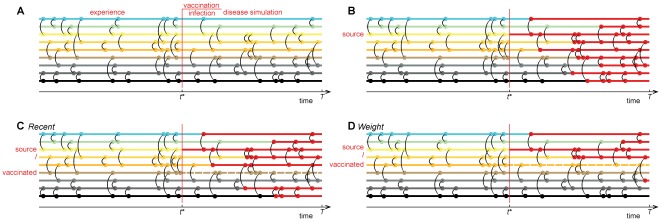
An illustration of a pictorial simulation of the immunization protocols. Panel A displays an artificial contact structure where each horizontal line represents an individual. The circles and vertical lines indicate the contacts. There are two regions, separated by half of the sampling time, one for learning (experience) and one for disease spreading. Panel B shows an example of a spreading process with 100% chance of contagion per contact, no recovery and no vaccination. Red lines represent infected individuals. In Panels C and D we see the same spreading event as in (B), but now, one individual is vaccinated by the *Recent* (C) or *Weight* (D) strategies. The ego indicates the vertex selected at random in the immunization protocol and the dotted line, its selected neighbor according to *Recent* or *Weight* strategy.

The two protocols we present in this paper use information from a random individual *I* in the community to find another individual to vaccinate who is more important in terms of disease spreading than *I*. The strategies are illustrated in Fig. 1C–D, for a hypothetical contagion of 100% transmission probability. In our first protocol, *Recent*, we iteratively asked a random individual *I* to name the most recent contact (of the sort that could transmit the disease in question) and vaccinated this person. The contact dynamics between two individuals has, at least in some circumstances, been observed to have a “bursty” dynamics—with alternating periods of activity and idleness [13]. The same pattern holds for the activity of individuals in the datasets we study in this work. The *Recent* protocol targets this type of temporal structure, and vaccinates individuals with a bias toward those currently in a period of heightened activity. In our second protocol, *Weight*, we iteratively asked a random individual *I* to name its most frequent contact since some time *t* in the past. This method seeks to vaccinate people who are, in general (or rather, over a longer time scale), more active than average. It is possible that one can make the protocols yet more efficient by choosing *I* as the last vaccinee to obtain chains of vaccinations [6], but in this work we use the above definitions to make the comparison with the well-known *NV* protocol transparent.

### Empirical datasets

We evaluate our strategies using four anonymized, empirical datasets extracted from electronic records of human interaction. Some of these datasets, which we present below, are more representative of the contact structures underlying disease transmission than other. The purpose of including lower quality datasets in the analysis is to see a wider spectrum of temporal effects (in the efficiency of the immunization protocols) as a ground for our general discussion.

The first dataset comes from a Brazilian online forum where male sex-buyers report and evaluate sexual encounters with female escorts (top-end prostitutes). This data spans 2,232 days, 16,730 people and 50,632 sexual contacts [14]. We connect a sex-buyer with an escort if they had at least one reported sexual encounter. We take the post's date as an estimate of the time of the encounter. Although this contact structure does not describe an entire sexual network, sexually transmitted infections (STI) can potentially spread over the contacts [15]. Nonetheless, qualitative conclusions (affected by the type of temporal and topological correlations present, not their magnitude) should be valid even if we use the data as a raw contact structure. Our second dataset records the proximity between patients in a hospital network. The data, described in detail in Ref. [16], cover 8,521 days and 295,107 patients living in the Stockholm region of Sweden. If two patients are on the same ward on the same day, we record that as a contact. In total, there are 64,625,283 such contacts that can be interpreted as potential spreading events of nosocomial disease [16]. The last two datasets come from online communications—one is the e-mail exchange dataset from Ref. [13], where 3,188 e-mail accounts were sampled over 83 days. An e-mail between two addresses is recorded as a contact. In total there are 309,125 contacts. E-mails to or from someone outside of the sampled e-mail accounts are ignored. This network captures some general features of human dynamics and is a representative structure for spreading of computer virus and information or opinions. More than that, however, its temporal structure gives a different type of behavior than the other datasets with respect to vaccination and we will use it as an example of such. The fourth dataset comes from an Internet dating community [17]. Various forms of communication between 29,341 members were recorded over 512 days, comprising a total of 536,276 contacts. Although the contacts in this community are precursors to romantic and sexual relationships (and thus potential disease spreaders), one can probably not draw any direct conclusion from it; rather, we include it as an example.

### Simulation of vaccination campaigns on empirical contact sequences

Contacts within a population have two functions in a vaccination campaign. First it is the connective structure that actually spreads the pathogen. Second, it is the basis for information from which we decide whom to vaccinate. At the time of the vaccination, we can only affect the disease spreading over contacts happening in the future, and base our decisions on contacts that have happened in the past. Therefore, in our simulations, we divide the sampling timeframe [0,*T*] into two periods [0,t*] and [t*,*T*] (where we chose t* as the time three-quarters of the contacts occurred) and use the first period only as the information source for the immunization protocol, and the second solely for the purpose of evaluation via disease simulation. In line with our stylized level of modeling, the vaccination is assumed to take place instantaneously at t*. This means that, in our study, the immunization program is assumed to occur at a time scale much shorter than that of epidemics, which is strictly speaking not the reality. Another motivation for this assumption is that the results would probably be qualitatively the same without it, so to avoid the complication of scanning different vaccination rates, we assume the rate is infinite. We also note that vaccines are usually distributed in batches that make the vaccination process pulse-like rather than continuous. Another assumption is that the disease is introduced into the system at the same time the vaccination program starts. While this is strictly speaking incorrect, it is feasible to assume that the vast majority of the population is uninfected at the time of the vaccination. A third unrealistic but simplifying assumption is that immunization is immediate and completely effective. Like the above assumptions, we make this one in order to keep the model mathematically simple and consonant with the rest of the literature. A more realistic model, with a non-zero probability of infection even though one is vaccinated, could be a topic for a deeper investigation, but would probably yield results similar to a rescaling of *f* (to smaller values, reflecting the occasional infection of a vaccinee).

### Upper bound on outbreak sizes

**Figure 2:**
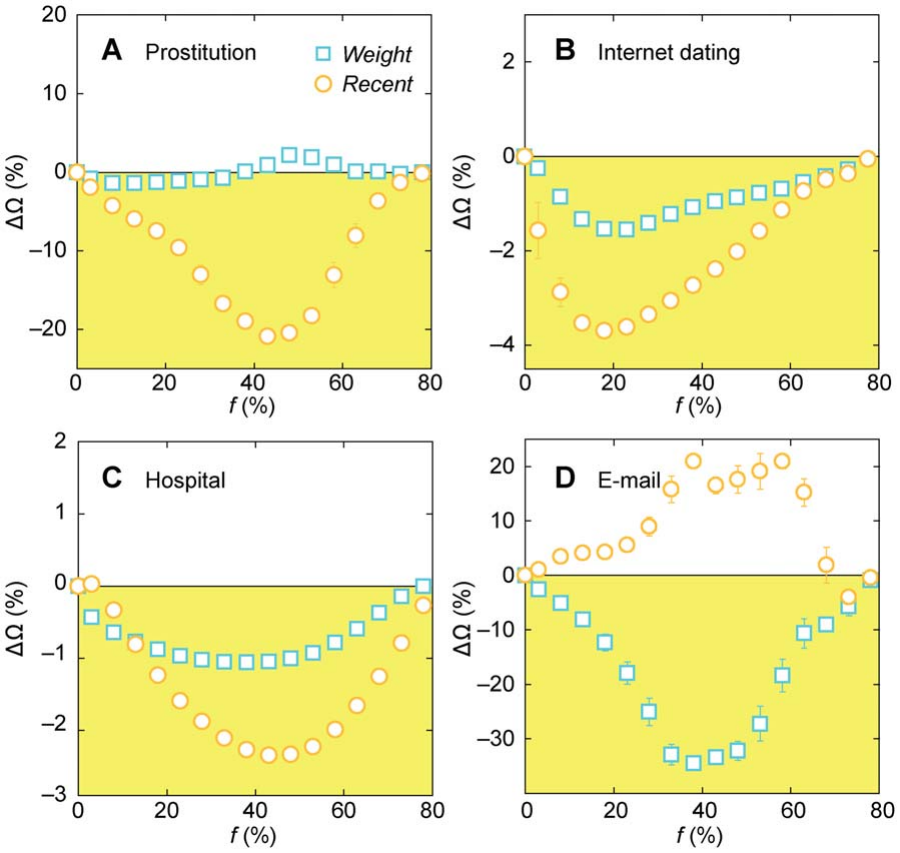
The performance of the *Recent* and *Weight* strategies relative to the *NV* method. The performance measure Ω is the upper bound of the outbreak size, given the temporal contact structures, averaged over all infection sources. The yellow regions indicate an improvement on *NV* (the more negative values, the better). The different panels correspond to the four different datasets. The error bars indicate standard errors over the set of infection sources.

In Fig. 2, we plot the performance of the strategies as a function of the fraction *f* of the population vaccinated. The performance measure is based on calculating Ω—the average upper bound of outbreak size (what one would get if all possible transmission events, where an infective person meet a susceptible, actually happens) in simulations as outlined above. We define Ω as the average over all vertices present in the contact set in the interval [t*,*T*] as infection sources. Ω is thus a measure for contact-sequences corresponding to the largest connected component in a static network—a common estimate of the severity of worst-case scenarios [18]. However, in contrast to the largest component size, Ω also includes temporal network effects such as that the disease can only spread from one vertex at time *t* via its edges active in the future of *t* [19], [20]. To quantify the relative benefits of the different strategies, we plot the fractional increase of Ω with respect to the *NV* method, ΔΩ. If, for example, ΔΩ = −10% the strategy in question decreases the upper bound of outbreak sizes by 10% relative to neighborhood vaccination. (The raw Ω-values can be found in Fig. S1 and a discussion in Text S1.) The prostitution, hospital, and Internet dating networks all yield similar results for ΔΩ; the curves for the e-mail data look drastically different (we will look further into why below). The relative advantage of *Recent* is strongest for the sexual contact network of Fig. 2A (with more than 20% improvement over *NV* at best). Our first conclusion is that ΔΩ is mostly negative—both *Recent* and *Weight* outperform *NV* for most datasets and fractions of the population vaccinated. *Weight* is typically better than *NV* (being about 20% better in the email network). *Recent*, on the other hand, performs worse than *NV* for the e-mail network but is better for the other contact sequences.

### Average outbreak sizes in dynamic simulations

To test the immunization protocols in a more realistic situation than the upper bound of outbreak sizes, we also run SIS simulations [1]. If we get qualitatively similar results from the SIS simulations that would be a strong indication that our results are stable. For example, the Susceptible–Infected–Removed (SIR) model, which is similar to SIS but does not, like SI, allow reinfections is in that sense intermediate between SI and SIS and would therefore (in practical situations) be expected to behave like an SI and SIS in agreement. In our simulations, a susceptible individual becomes infected upon contact with an infected with a probability λ. We let the infected stage last a fixed duration δ. We go through all unvaccinated vertices as sources of infection and simulate the disease spread within the interval [t*,*T*]. It might thus happen that the source is only present in the data before t*, in which case it would certainly not infect anyone else.

**Figure 3:**
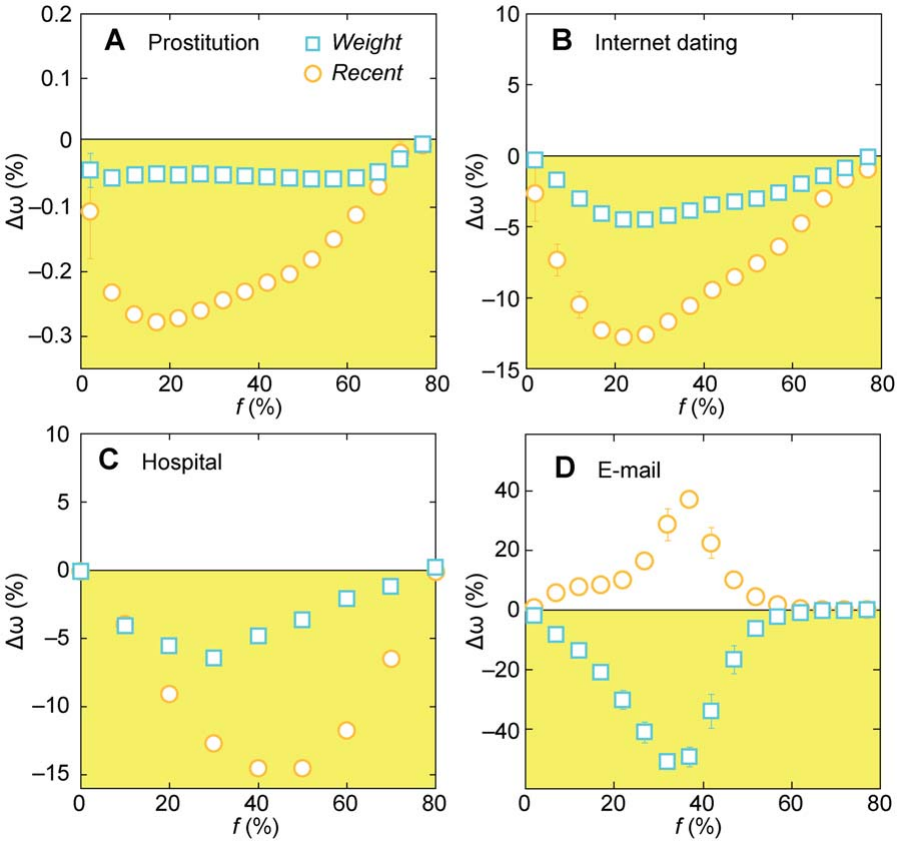
The performance of the *Recent* and *Weight* strategies relative to the *NV* method for a dynamic, SIS-type disease simulation. The performance measure in this case is the average outbreak size ω (total number of infected individuals) in a SIS simulation with a per-contact transmission probability λ = 0.25 and a duration δ of the infected stage of three weeks. Just like Fig. 2, the vaccination is more efficient, relative to *NV*, the lower Δω is. The error bars correspond to the standard error calculated over all unvaccinated vertices as infection sources and 1000 runs of the vaccination and SIS simulation per source.

The first quantity we look at for these simulations (see Fig. 3, which shows results for SIS) is the average fraction of individuals that is infected at least once ω (averaged over all unvaccinated individuals as infection sources and 1000 random seeds) as a function of *f*. (We plot the raw ω-values in Fig. S2, and discuss them in Text S1.) For this plot we use the parameter values λ = 0.25 and δ = 3 weeks. We choose this transmission probability to roughly reflect realistic diseases (for example, less contagious than chlamydia [21], more than HIV [22]), and short durations to capture dynamic effects of the finite duration of diseases. Since the datasets are limited in time, such effects would vanish if δ was much longer. The SIR (Susceptible–Infected–Removed) model with the same parameter values yields rather similar curves—the skewed distribution of activity in these datasets means that the probability of re-infection (the difference between SIS and SIR) is significant only for the comparatively small group of most active individuals.

The curves in Fig. 3 are strikingly similar to those in Fig. 2. Only the magnitude of the differences varies—for the prostitution Δω (Fig. 3A) is consistently smaller than ΔΩ (Fig. 2A); for the other three datasets, the difference in performance is larger (about 15% improvement for the Recent strategy in the Hospital and Internet dating networks and more than 30% for the Weight strategy for the email network) for the SIS simulations in comparison to the worst-case scenario measure, Ω. One explanation for the small differences in the prostitution data is that about three-quarters of the contacts occur only once. Our strategy *Recent* can eliminate a worst-case scenario by finding people involved in these rather rare recurring contacts; however, for the average outbreak sizes measured in the SIS simulations, the chance of an outbreak is so small, that the ω-values do not differ much.

### Relative advantage of strategies as a function of infectivity and duration of the infective state

**Figure 4:**
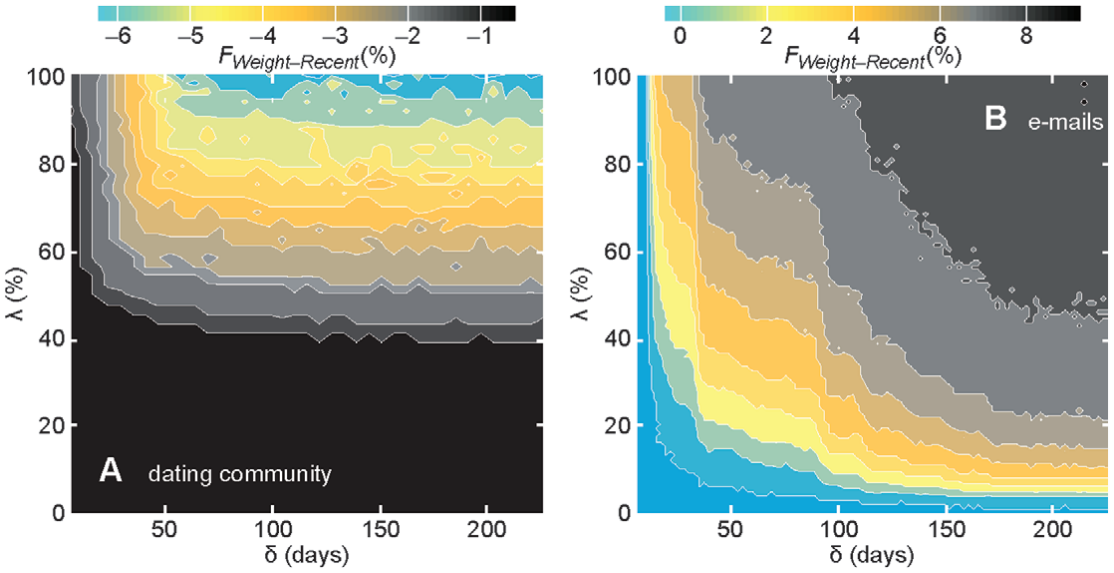
The relative performance of *Recent and Weight* over the SIS model's parameter values. We measure a quantity *F_Weight–Recent_* that is large if an SIS outbreak, on average, is better stopped by *Weight* than *Recent*. More specifically, we calculate which immunization protocol that would most efficiently (in terms of the lowering the number of infection events during the simulation) stop an infection starting at vertex *i*, and average it over all *i*. *F_Weight–Recent_* is the deviation from a neutral situation of *Weight* and *Recent* being most effective for an equal fraction of vertices. For every parameter value, we use all unvaccinated vertices as infection sources and 100 runs of the immunization protocol and disease simulations. In this plot, we use *f* = 20%. The dating-community data (A) behave qualitatively like the prostitution and hospital contact data.

**Figure 5:**
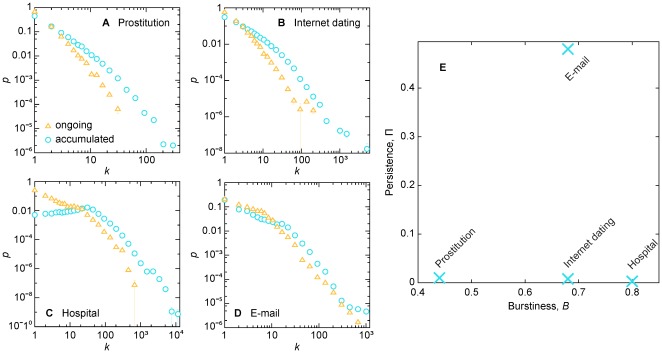
Degree distributions of the empirical datasets. In panels A–D, we plot the probability density *p* as a function of degree *k*. We plot results both for the accumulated network of all contacts and averages of three networks of ongoing contacts (defined by all edges that, at a certain time *t′*, a contact over all edges have happened and will happen again). We choose *t′* as when a quarter, half and three quarter have happened. In panel E, we show the values of two types of temporal statistics of the datasets—the persistence (which separates the e-mail data from the rest) and burstiness.

We continue our analysis of how the vaccination affects the average outbreak sizes in stochastic simulations by looking at the response of ω to the model parameters λ and δ. In this analysis, we keep *f* = 20%—a value close to where the choice of immunization strategy makes most difference. In addition, the four datasets in this analysis fall into two classes where the e-mail data exhibits a unique behavior and the three others are similar to each other. We let the smallest dataset of this category—the Internet dating network—represent the whole class. To evaluate the strategies, we go through all the unvaccinated individuals as sources of the epidemics, apply the immunization protocols, and calculate, for a pair of immunization strategies A and B: out of 100 runs of the SIS model, how many times strategy A outperforms strategy B. In Fig. 4, we present the deviation in percent *F_Weight–Recent_* from a scenario where the strategies are equally successful (other combinations of strategies, including *NV* can be found in the Fig. S3 and a discussion in Text S1). The main conclusion is that the observation from Fig. 3 holds throughout the (λ,δ) parameter space—*Weight* is the best strategy for the e-mail data; *Recent* is the best for the others. In the small λ and small δ limit, the disease will die out soon whether someone has been vaccinated or not. This explains why the smallest deviations, both in Fig. 4A and B, occurs for the smallest (λ,δ)-values. Then, if we focus on the dating community in Fig. 4A, there is a dramatic change in *F_Weight–Recent_* as λ exceeds 50% for δ>40 days. This is related to an epidemic threshold that, despite the skewed degree distributions (Fig. 5A–D), is rather clear for this type of data [15]—for λ>50%, a disease can spread to a finite fraction of the population, and the immunization protocols do make a difference for this dataset. Furthermore, if one varies δ, *F* responds in a highly non-linear manner. If the duration of the infection is long enough, the benefits of the strategies are similar, but for diseases short in duration, *F* changes rapidly with δ. For the e-mail data there is a similar plateauing δ-dependence of *F*, but λ-dependence is closer to zero, rather than an intermediate value.

### Model of artificial contact sequences

From the above studies we may conclude that, regardless of the type of the disease, *Recent* is the best strategy for the prostitution, Internet dating, and hospital proximity data, whereas *Weight* is the better strategy for the e-mail dataset. Why? *Recent*, *Weight* and *NV* all handle topology in the same way, in the sense that the vaccinee is chosen from the same neighborhood, so any difference in efficiency probably comes from the temporal characteristics of the activity between two persons. This is further corroborated by the fact that the degree distributions—both of the accumulated contact network and the network of ongoing contacts (that at a specific point has happened and will happen again)—are qualitatively similar for all four datasets (Fig. 5A–D). A candidate explanatory temporal structure is *burstiness* [23], the phenomenon that human activities of some specific type often are grouped in time. It turns out that all our datasets have fairly high burstiness (measured by a quantity presented in the Methods section) and it cannot separate the e-mail data from the others (Fig. 5E). One aspect that sets the e-mail data apart, however, is that the edges are fairly persistent (measured by the fraction of edges that is present both in the first and last 5% of the contacts). In a situation like the e-mail data where the overall activity is rather uniform, the activity of the more distant past is more reliable in predicting future activity. The Internet dating, hospital, and prostitution networks are more dynamic, with individuals entering and leaving the system (here, the persistence about 50 times less than the e-mail data). The trend in the Internet dating community is on the increase in terms of system-wide activity level, whereas the hospital and prostitution data show a more quasi-stable behavior where individuals enter and leave the system at a more constant pace. If we assume a situation where in terms of activity identical users come and leave the system at equal rates, the users most recently seen to be active are also the ones most likely to be active in the near future, and thus the ones most urgently requiring vaccination. This helps us to understand why *Recent* is the best strategy for Internet dating, prostitution, and hospital proximity network.

**Figure 6:**
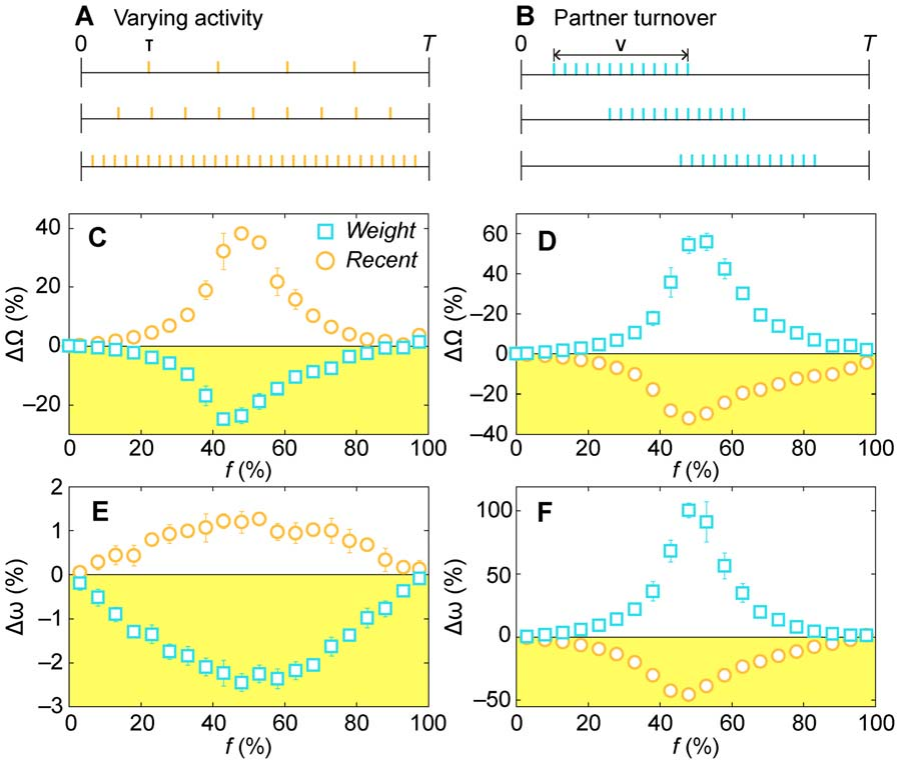
Evaluating the performance of the vaccination strategies for different types of temporal correlations. In A and B, we illustrate the models that encode the different temporal contact structures. In the varying activity model (A), the first contact along an edge happens at time *t_s_* after the beginning of the simulation and then subsequent contacts happen with a time interval *t_s_*. In the other, partner turnover, model (B), an edge becomes active with uniform probability in time the interval [0,*T*−*ν*]. The edge is active for *ν* time steps with one contact per time step. Panels C and D show the worst-case scenario, Ω, and panels E and F show the average outbreak sizes in the SIS model. The networks used in C and E follow the temporal profile shown in panel A; panels D and F follow the profile illustrated in panel B. The underlying network topology is the Erdős-Rényi model, which has a minimum of structural bias.

To put the arguments above on a more solid footing, we construct two models of contact patterns capturing these two temporal structures (see illustrations in Figs. 6A and B). In both these models, the network structure is purely random (details in the Methods section) to ensure that all of the effects we observe are temporal. In the first model, which captures *varying activity* (the *VA* model), we let communication over an edge (a connected pair of vertices in the network) take place at intervals of τ, a value drawn from a uniform distribution, until time reaches *T*. The second model embodies the birth and death of relationships—each edge is active for a fixed duration (Δ*t* time steps, with one contact per time step), but the starting time is random. We call this model the *partner turnover* (*PT*) model.

In Figs. 6C–F, we plot the results from our simulations of the contact pattern models. These simulations, which investigate both worst-case scenarios (Figs. 6C–D) and average outbreak sizes in the SIS model (Figs. 6C–F), confirm that temporal structure can create the different efficacies of the immunization protocols. For the VA model, since the neighbor to vaccinate is chosen in proportion to weight, the chance of picking a highly active individual is higher with the *Weight* strategy than *NV*. If *Recent* is applied to the VA model in our range of parameters (relatively large t*), there is a heightened chance that the latest contact is one with a small τ that will not recur (Text S1), which makes *Recent* perform worse than *NV*. For the PT model, if an edge recently had some activity, chances are high that it will be active again soon. *Recent* is designed to find such recently active edges, so it logically works better than *NV* in this situation. If there are relationships that are over in the PT model, then *Weight* will pick one of those. This is clearly counterproductive compared to sampling recently active (like *Recent*), like the *NV* strategy, just choosing a random neighbor. The best strategies for each of these artificial networks improve the *NV* protocol by 10–40%. One can show analytically (see Text S1 and Fig. S4), that using the accumulated degree as a proxy for the importance of the vaccinated vertex, *Recent* performs better than *NV*, which performs better than *Weight* for the partner turnover model, and *Weight* performs better than *NV*, which performs better than *Recent* for the varying activity model, for most realistic parameter values.

## Discussion

In this paper, we propose two immunization protocols seeking to exploit both the temporal and topological contact structures. We limit ourselves to protocols that are practically realizable (admitting that the problem formulation is very simplified compared to the politics of real vaccination campaigns). Our strategies utilize both temporal and topological contact structure, and we show that they are more effective than the neighborhood vaccination protocol (that uses only static topological information). The two strategies are based on sampling individuals at random, asking them “Who where you in contact with most recently [in such a way that disease could have spread]?” (the *Recent* strategy) or “Who were you in contact with most often the last *X* months?” (the *Weight* strategy), and then vaccinating the named individual. In this paper, we study the long-term limit of *X*, so that the *X* months cover all the datasets before the vaccination time. To clearly observe the effects of the temporal structure (in contrast to the network topology), we limited ourselves to straightforward extensions of the neighborhood vaccination protocol. One can imagine extensions like recursive, chained applications of these protocols (cf. Ref. [6]), or a Bayesian approach where one scan the patterns predicting future contacts for the ones optimizing vaccination.

We test the strategies on four empirical datasets of contacts. These are primarily intended as examples to prove the main point that simple local vaccination strategies *can* exploit temporal network structure (and leave it to future studies to devise vaccination strategies for general temporal-network structures). Two of these datasets represent possible pathways for real epidemics (a sexual network of Internet-mediated prostitution and a proximity network of patients in a hospital system). The other two datasets come from online communication (where, presumably, an edge means a high chance for an offline social tie, but the temporal contact structure is probably not so correlated with the offline contacts). We split the sampling times of the data into two parts, the first where the individuals experience the world, and the second where an epidemic spreads via the contacts. In this work, we set this breaking point at three quarters of the sampling time, but this choice is rather arbitrary, setting it to half or 90% of the sampling time gives qualitatively the same results (not shown). Furthermore, we see that the conclusions are qualitatively unchanged with shorter sampling periods (we truncate half of the time series and observe the same response). Like all other empirical datasets constituting subsets of human contacts, our data can have biases from the sampling procedure. That a person disappears from the prostitution data does not mean the person become inactive. At the moment there are no theories for how to compensate for such effects in temporal networks (as there are for static networks [24]), so we make the assumption that the structures are not artifacts of the sampling (which is to some extent corroborated by the facts, mentioned above, that results are insensitive to truncating the data and moving of the breaking point). If one would consider the same scenario with much longer data sets, so long that the behavior of individuals have time to change, then the oldest information would not be worth much for predicting important people to vaccinate in the near future. Symmetrically, the selection of people to vaccinate at present will not matter for outbreaks in the far future. To generalize these two cases, events far from now cannot affect, or be affected, by the temporal network structure, but at worst *Recent* and *Weight* will perform as random vaccination.

In contrast to other vaccination simulations [3], [6]–[9], we do not assume that contact patterns are the same before and after vaccination. In these other studies, the network that will transmit the disease after vaccination is already used as a basis for identifying individuals to vaccinate. In this respect, our approach is more strict and realistic compared to the above-mentioned studies.

As it turns out, the *Weight* strategy outperforms *Recent* and *NV* for the e-mail data while *Recent* is the most efficient method for the other three datasets. This tells us four things. First, there is enough temporal structure in the contact patterns for our protocols to be effective. Second, the optimal choice of immunization protocol can be dependent on the specific contact structure of a disease. Third, in the more realistic networks that we investigate *Recent* is the better strategy (although the datasets are so few that such a generalization should be taken with a grain of salt). Fourth, the temporal correlations of these more realistic networks are relatively short. After a closer look at the temporal structures separating these datasets, using models of contact dynamics (where one can control the temporal structure,), we argue that a turnover of relationships promotes the efficiency of *Recent*. A similar result is Koopman *et al.*'s finding that short-term fluctuations are more important than long-term changes for HIV transmission [25]. In general, temporal-network based methods can be more efficient than the static-network approaches within a time window of the size of the correlations in the data. Too far into the past or future both *Weight* and *Recent* will converge to *NV*. *Weight*, in contrast, is most efficient when the ties between individuals overlap strongly in time, but there is a broad distribution of contact rates over those ties. These conclusions seem to hold irrespective of the degree distribution of the aggregated network (as we test both on the skewed, fat-tailed empirical networks and model network that have degrees distributed by the narrow Poisson distribution). Still, it could of course be the case that real systems have other temporal structures, which illustrates that we need future studies both to characterize the temporal-network structure of real-world contact structures and to propose vaccination strategies that exploit these structures. In a real implementation, the naming of a person by another, picked at random, could be erroneous both when it comes to pointing out someone that has been in such a close contact that a disease could have spread, and assessing the order (for *Recent*) or intensity (*Weight*) of the contacts. On the other hand, if there are large errors in the latter, time-related assessments, then *Recent* and *Weight* will effectively approach the *NV* protocol. If, in addition, there is a significant inaccuracy in the assessment of whom that has been close enough for contagion, then all three protocols—*Recent*, *Weight* and *NV*—approach random vaccination. Most practical vaccination campaigns are voluntary. Assuming voluntary vaccination is not primarily guided by risk-awareness, it probably comes close random vaccination. So *Recent* and *Weight* would at worst, in the case there is no information to utilize, perform like voluntary vaccination.

We mentioned in the Introduction that vaccination is to be taken in the most general sense, as reducing the risk a specific individual gets and transmits a disease. When it comes to practical vaccination of real infectious disease, Hepatitis B is perhaps the pathogen that fits our protocols best for two reasons [26]. First, its primary contagion pathways are sexual contacts and injecting drug use, so contacts that could transmit the disease are easily recognizable. This increases the accuracy of the naming step of the protocols, and thus their efficiency. Second, it has effective vaccines. One can also imagine HIV prevention as an application, but vaccination should then be read as counseling, perhaps in combination with antiretroviral prophylactics. Another type of clinical practice that could be improved by our protocols is the partner treatment of diseases like chlamydia, gonorrhea and trichomonas, where an infected patient can get medication for a partner without the partner having to be examined [27]. If the patient has more than one sexual partner, the choice of whom to include in the partner treatment could be guided by the most recent or the most frequent partner since some time into the past. In a wider perspective, a related sampling procedure of our vaccination protocols is contact tracing [28], where one tries to sample people within an epidemic outbreak by having everyone testing positive to report their previous contacts and testing these. In case one does not want to make a complete sampling of the former contacts of infected individuals, a slightly modified *Recent* or *Weight* could be used to set the priority of whom to call for testing.

At a fundamental level, the fact that, no less than the topology, temporal structures can influence the efficiency of immunization protocols must be more thoroughly understood. Similar problems arise in other areas where one seeks to limit a spreading phenomenon dependent on contact dynamics such as diseases in wild or domestic animals, e-mail viruses, or computer worms [29]. We anticipate more research in this direction.

## Methods

### Disease-spreading simulation

All the datasets we use can, mathematically, be represented as lists of *contacts* (*x_i_*,*y_i_*,*t_i_*), *i* = 1,…,*C*. Each triple represents a contact between individual *x_i_* and *y_i_* at time *t_i_*. We can assume that a contact list is ordered such that *t_i_*≤*t_i_*_+1_ for all *i*. Without loss of generality, we set *t*_1_ = 0. *T*, the total sampling time, is thus simply *t_C_*. Let *N*(*t*) be the number of vertices at time *t* and *N* (without argument) denote *N*(*T*).

We divide the sampling time into two parts [0,t*] and [t*,*T*] where t* = *t_x_* = *t*_3*C*/4_. At time t* we both vaccinate the population and start the disease. We choose one vertex among the entire unvaccinated population (even if their last contact is before, or first contact after, t*), with uniform randomness, as an infection source. The immunization protocols use the experience from the interval [0,t*], but no information whatsoever about the interval when the epidemics is unfolding [t*,*T*]. The results in the paper are qualitatively rather insensitive to the choice of *x*. However, if *x* is too small the information the protocols can act upon is smaller and naturally their performance worse. If *x* is too large then the time for the disease simulations get too short. Our results are roughly speaking stable in the interval 50%<*x*<90%, so we settle for *x* = 75% as a round number.

At time t* we choose *Nf* individuals to vaccinate (where *f* is a control parameter setting the fraction of the population to vaccinate). The flow chart of the simulation is:

1. With uniform probability, pick an individual *i* among the *N*(t*) individuals present in the data at this time.
2. Pick a neighbor *j* of *i*, either the most recent contact of *i* (the *Recent* protocol) or the most frequent contact in the interval [t*−*X*, t*], 0≤*X*<t* (*Weight*), or any contact in this interval with uniform probability (*NV*). For simplicity, we use *X* = t* in this paper.
3. If such a vertex *j* exists and is not vaccinated, then vaccinate *j*.
4. If *Nf* vertices are not yet vaccinated, go to step 1.

One run of the SIS disease simulation starts by marking one source vertex as being infected, and all other vertices marked as susceptible. Then we go through all contacts (*x_i_*,*y_i_*,*t_i_*), 3*C*/4<*i*≤*C*, and if *x_i_* (*y_i_*) is infected, but not *y_i_* (*x_i_*), then, with a probability λ, *y_i_* (*x_i_*) becomes infected. After a time δ, an infected vertex becomes susceptible again. Our key quantity is ω—the total fraction of infected vertices at time *T*. When we study the average upper bound of outbreak sizes—technically equal to the outcome of a SIS simulation with λ = 1 and δ = ∞—we use the symbol Ω (instead of ω) for the average number of individuals that can be reached by successive contacts from the source. To calculate ω and Ω, we average over all (1−*f*) *N* unvaccinated vertices as infection sources and 1000 independent runs of the immunization protocol and disease propagation.

### Burstiness

Burstiness is a feature typical for time lines of events in the life of a human. If you, for example, look at the times a person sends email, they are typically grouped into periods of intense activity, “bursts,” with few contacts in between. We follow Ref. [23] and define a measure of burstiness as the coefficient of variation of the times τ between events—*B* = (σ_τ_−*m*_τ_)/(σ_τ_−*m*_τ_), where σ_τ_ is the standard deviation of τ and *m*_τ_ is the mean. B takes values between −1 and 1 where −1 indicates a completely regular signal, 1 is a maximally bursty signal and 0 represents neutrality.

### Models of contact dynamics

To elucidate the effects of the temporal structure on the immunization protocols, we use two generative models of contact sequences. The network topologies of these simulations are the same—an instance of an Erdős–Rényi model [30] with 1000 vertices and 2000 edges. The idea is to generate an underlying network topology with as little structure as possible, to test the hypothesis that the relative performance of *Recent* and *Weight* are more dependent on the temporal, than the topological, aspects of contact structure. Given the topology, we associate every edge with a set of contacts generated by one of two methods. For the first method (the varying activity model), we draw a random number τ with uniform probability in the interval [0,*T*]. Then we let the contacts over the edge take place at times τ, 2τ, …, *n*τ, where *n* is the largest number such that *n*τ<*T*. In the other method, the partner turnover model, the contacts take place over Δ*t* consecutive time steps. The starting time for this burst of contacts, *t_s_*, is a random variable drawn with uniform probability from the interval [0, *T*−Δ*t*]. We use *T* = 10,000 and Δ*t* = 250.

### Ethics statement

The data about patient flow was approved by the Regional Ethical Review Board in Stockholm (Record Number 2004/5:8). No informed consent was obtained but all data were analyzed anonymously.

## Supporting Information

Figure S1 **The upper limit of the outbreak sizes** ***Ω*** **for our two vaccination protocols, neighborhood vaccination and an unbiased vaccination of the** ***f*** **individuals.** Different panels are for different datasets (corresponding to Figs. 2 and 3 in the paper). The points are averaged over all unvaccinated vertices as infection sources and 1000 realizations of the vaccination scheme and disease simulation per infection source. Error bars display standard errors.
(TIF)

Figure S2 **The average outbreak size** ***ω*** **for our two vaccination protocols, neighborhood vaccination and an unbiased random vaccination of the** ***f*** **individuals.** The parameter values are λ = 0.25 (λ is the per contact transmission probability) and a duration δ = 3 weeks of the infected stage. Different panels are for different data sets (corresponding to Figs. 2 and 3 in the paper). The points are averaged over all unvaccinated vertices as infection sources and 1000 realizations of the vaccination protocol and outbreak simulations. Error bars display standard errors.
(TIF)

Figure S3 **The performance of the** ***Recent*** **and** ***Weight*** **strategies relative to the** ***NV*** **model for an SIS disease simulation.** The performance measure *F*_A–B_ shows which strategy is most efficient (per infection source) relative to a neutral situation where the strategies A and B are equally efficient (cf. Fig. 4 in the paper). For every parameter value, we use all vertices as infection sources and 100 runs of the vaccination protocol and disease simulations. Our other datasets (from prostitution and hospital contacts) behave qualitatively like the dating-community data (A).
(TIF)

Figure S4 **Illustration of quantities for the discussion of the varying activity model.**
(TIF)

Text S1 **Supporting statistics and analytic derivation of the contact model's behavior.** In this text, we discuss some additional statistics (the raw values of upper bounds on outbreak sizes and raw values on average outbreak sizes in SIS simulations) that give the same conclusion as the figures in the main article but from different angles. We also include an analytic derivation of the response of the vaccination protocols to the two models of contact patterns along an edge.
(DOCX)
