# Supplementary figures and images for: Exploiting Temporal Network Structures of Human Interaction to Effectively Immunize Populations
Source: PLoS One. 2012 May 7;7(5):e36439. doi: 10.1371/journal.pone.0036439 (PMC3346842; doi:10.1371/journal.pone.0036439)

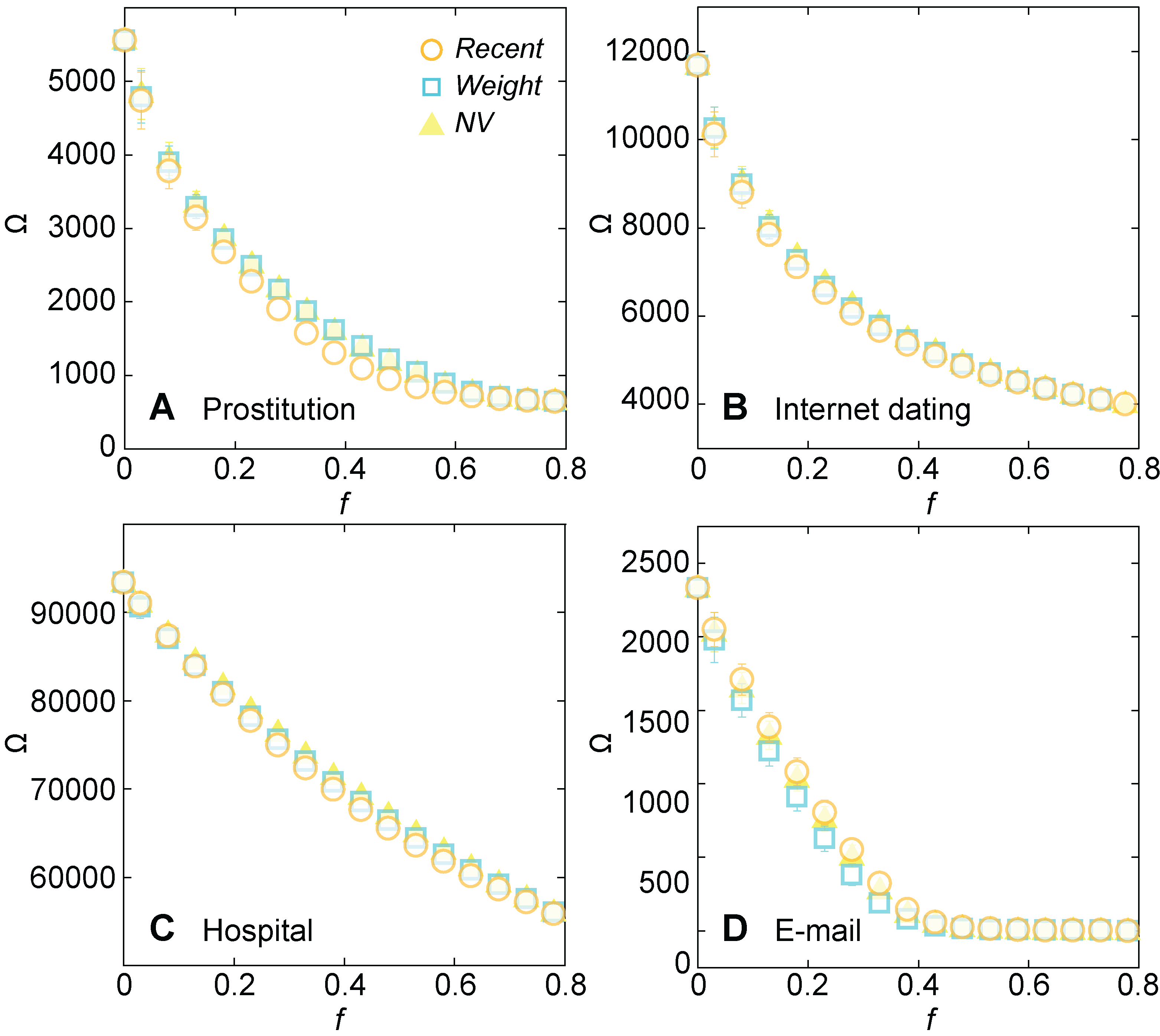

Supplement: Figure S1 — The upper limit of the outbreak sizes Ω for our two vaccination protocols, neighborhood vaccination and an unbiased vaccination of the f individuals. Different panels are for different datasets (corresponding to Figs. 2 and 3 in the paper). The points are averaged over all unvaccinated vertices as infection sources and 1000 realizations of the vaccination scheme and disease simulation per infection source. Error bars display standard errors. (TIF) [file pone.0036439.s001.tif]

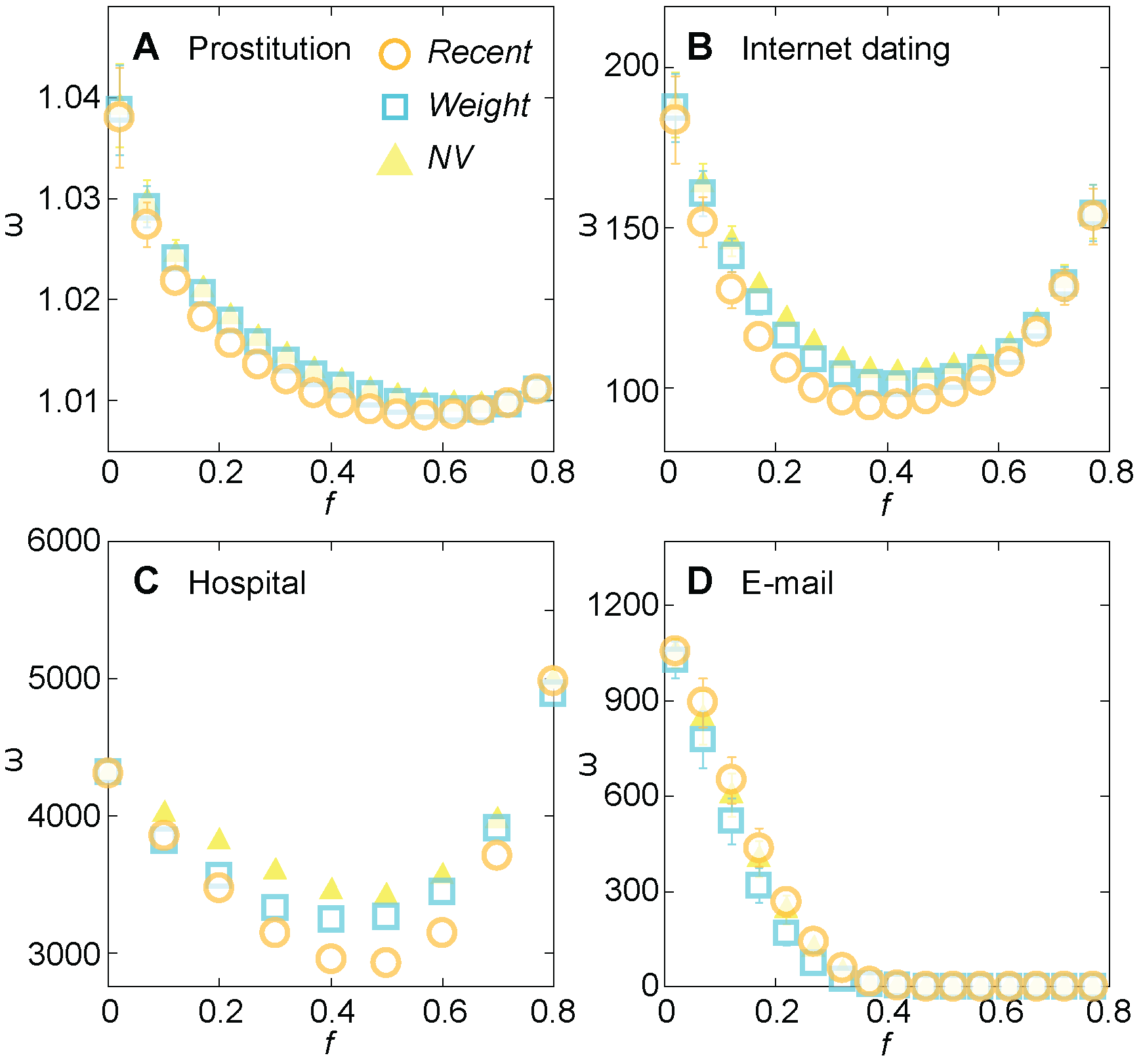

Supplement: Figure S2 — The average outbreak size ω for our two vaccination protocols, neighborhood vaccination and an unbiased random vaccination of the f individuals. The parameter values are λ = 0.25 (λ is the per contact transmission probability) and a duration δ = 3 weeks of the infected stage. Different panels are for different data sets (corresponding to Figs. 2 and 3 in the paper). The points are averaged over all unvaccinated vertices as infection sources and 1000 realizations of the vaccination protocol and outbreak simulations. Error bars display standard errors. (TIF) [file pone.0036439.s002.tif]

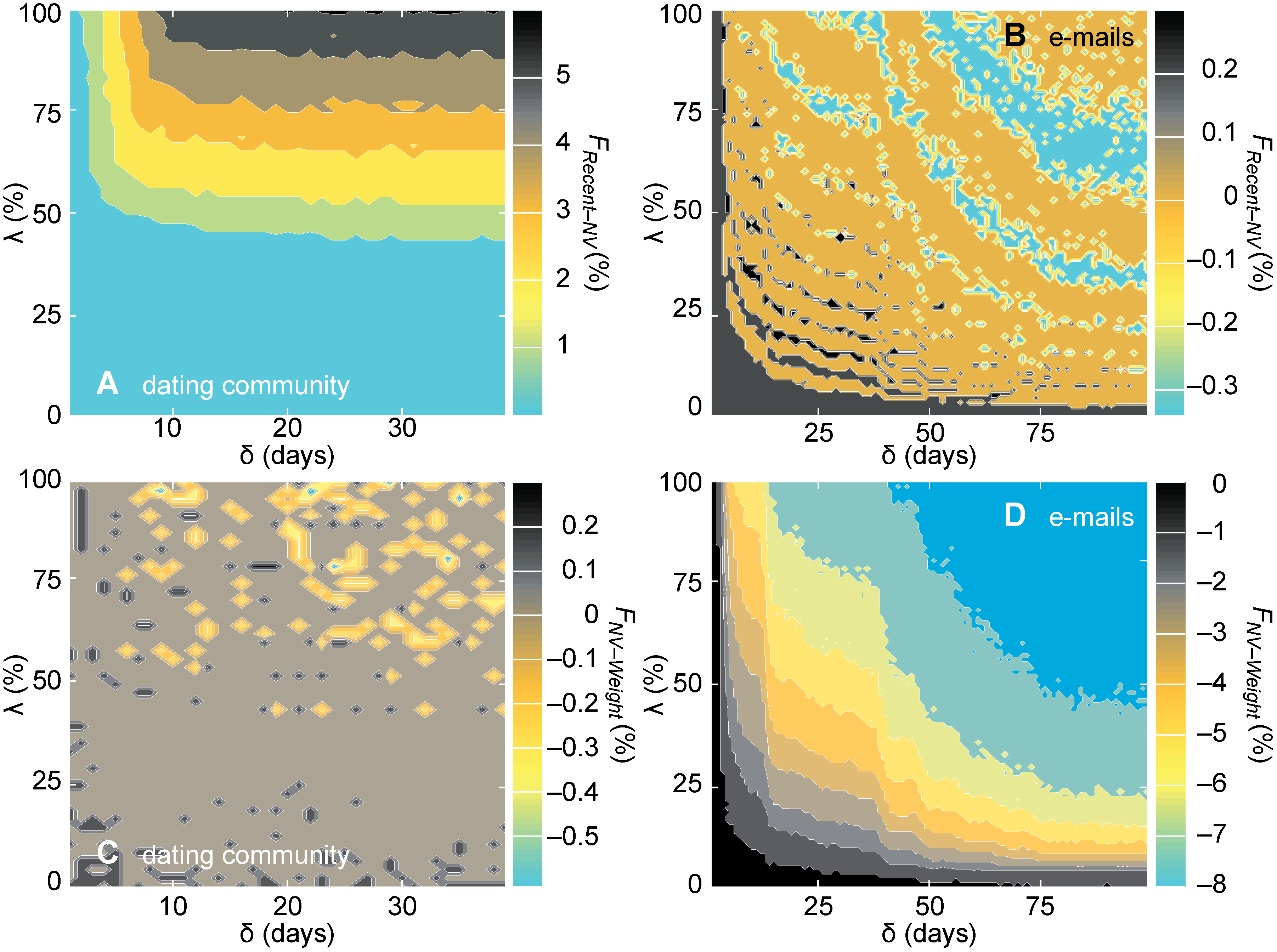

Supplement: Figure S3 — The performance of the Recent and Weight strategies relative to the NV model for an SIS disease simulation. The performance measure F A–B shows which strategy is most efficient (per infection source) relative to a neutral situation where the strategies A and B are equally efficient (cf. Fig. 4 in the paper). For every parameter value, we use all vertices as infection sources and 100 runs of the vaccination protocol and disease simulations. Our other datasets (from prostitution and hospital contacts) behave qualitatively like the dating-community data (A). (TIF) [file pone.0036439.s003.tif]

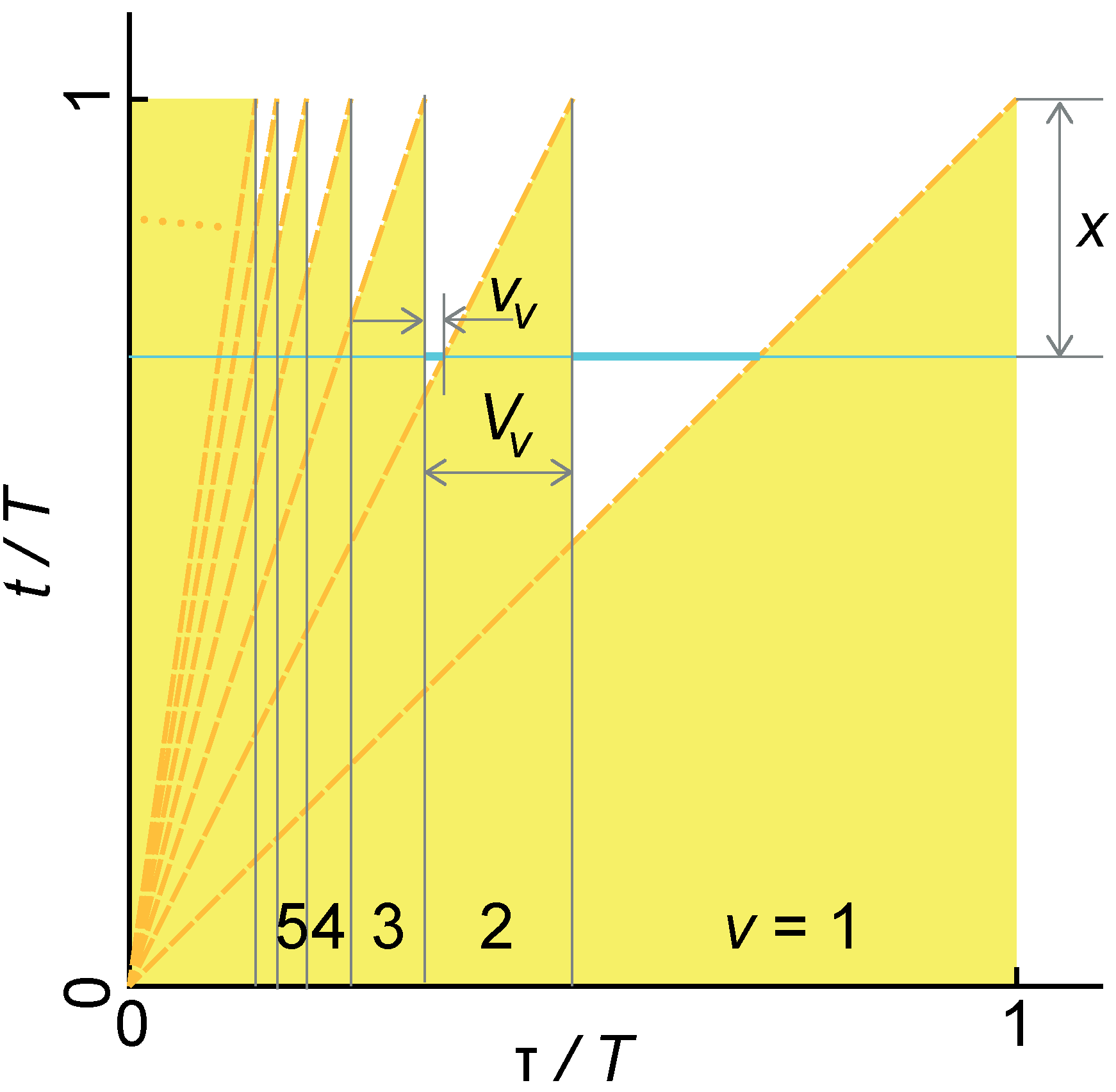

Supplement: Figure S4 — Illustration of quantities for the discussion of the varying activity model. (TIF) [file pone.0036439.s004.tif]
