# Supplementary material for: Exploiting Temporal Network Structures of Human Interaction to Effectively Immunize Populations
Source: PLoS One. 2012 May 7;7(5):e36439. doi: 10.1371/journal.pone.0036439 (PMC3346842; doi:10.1371/journal.pone.0036439)
Supplement: Text S1 — Supporting statistics and analytic derivation of the contact model's behavior. In this text, we discuss some additional statistics (the raw values of upper bounds on outbreak sizes and raw values on average outbreak sizes in SIS simulations) that give the same conclusion as the figures in the main article but from different angles. We also include an analytic derivation of the response of the vaccination protocols to the two models of contact patterns along an edge. (DOCX) [file pone.0036439.s005.docx]

Supporting Information

**Exploiting temporal network structures of human interaction to effectively immunize populations**

Sungmin Lee^1^, Luis E. C. Rocha^1^, Fredrik Liljeros^2^ and Petter Holme^1 2^ *

^1^ *IceLab, Department of Physics, Umeå University, 90187 Umeå, Sweden*

^2^ *Department of Sociology, Stockholm University, 10691 Stockholm, Sweden*

* Corresponding author:

Petter Holme

Department of Physics

Umeå University

90187 Umeå, Sweden

Phone: +46 90 786 5540

Fax: +46 90 786 6673

E-mail: petter.holme@physics.umu.se

In this Supporting Information, we show some auxiliary plots to provide a more comprehensive picture (although not much more information) than in the paper.

**Raw Ω values**

In Fig. S1, we plot the raw values of the maximal outbreak sizes, Ω, corresponding to Fig. 2 in the paper. If no vertex is vaccinated, the epidemics can reach large parts of the networks, even considering temporal effects. There is thus a connected component spanning a large fraction of the population, even considering temporal effects [1] (which is a prerequisite for diseases to be able to spread). We also note that the random vaccination protocol performs dramatically less well than the others. The fact that *Weight* and *Recent* never are far from *NV* suggests that the topological effects dominate the temporal ones in our four data sets. The advantage over *NV* is stronger in topologically more homogeneous contact networks, like those behind diseases that require relatively long exposure [2]. Our best protocol (given the contact structure) will on the other hand never be worse than *NV* and since it is no more costly, we believe it is a better choice in practice.

**Raw ω values**

Fig. S2 corresponds to Fig. 3 as Fig. S1 corresponds to Fig. 2. Thus, Fig. S2 shows the average outbreak size in the SIS model for a per-contact transmission probability of 0.25 and a duration of the infection of 21 days. One observation is that, for the hospital data, the relative disadvantage of a random vaccination is smaller than in Fig. S1, while the advantage of *Recent* over *NV* is simultaneously larger. It may thus be the case that real epidemics accentuate the advantages of the temporal protocols. Another feature of ω that may seem counterintuitive at first is that it can increase for large enough *f* values. The reason for this is that the vertex to vaccinate is chosen from among the vertices present in the data at time *t**, whereas the infection source is selected from among any non-vaccinated vertices. If *f* is small, there is a fair chance that the vaccinated vertex will not appear in the data again after *t**, and thus the disease cannot spread beyond the source. If *f* is large enough, on the other hand, the infection source is likely a vertex that has not yet appeared in the data, and thus more likely a vertex that can start a substantially large outbreak. An alternative way of running the simulation would be to choose a source among all the *N* vertices, in which case this effect would disappear.

**Performance of *Recent* and *Weight* vs. the *NV* protocol**

Fig. S3 shows plots corresponding to Fig. 4, where we compare the efficiency of *Recent* and *Weight* with the *NV* protocol over a large part of the SIS-model’s parameter space. The plots display the fraction of infection sources for which a certain vaccination strategy is, on average, best. We plot the relative difference between two strategies, subtracting 50% to make 0 represent neutrality. We see that the results from Fig. 3 (that *Recent* is better than *NV* for dating-community-type data, and *Weight* is better than *NV* for the e-mail contacts) are accentuated for long disease duration and high infection probabilities. For the dating community data, the *NV* and *Weight* strategies are almost identical see (Fig. S3C).

**Analytic approximate solutions of the contact models**

**Overview**

Here we present our analytical treatment of the models of contact sequences with varying activity levels and a turnover of partners discussed in the main text (and illustrated in Figs. 5a and b in the manuscript). For both these models, the structure over which the vertices interact is an uncorrelated random graph, or Erdős–Rényi model [3]. Starting from an empty graph of *N* vertices, we add *M* edges. An edge is drawn by selecting two vertices randomly, and is accepted if it is not a self-edge (between a vertex and itself) or has already been added to the graph. From the generated graph, we go over all its edges and add contacts by two different schemes. In the first scheme, in which model a varying activity level for each edge (*i*, *j*), we draw a random time interval τ from a uniform distribution between 0 and *T*. Contacts over this edge will then take place at times *τ*, 2*τ*, … , *nτ* , where *n* is the largest number such that *nτ* < *T* . In the second scheme, modeling partnerships as they form and break, a contact is active for fixed time interval $\left[ t_{s},t_{s}+\Delta t \right]$ where *t_s_* is chosen with uniform probability in the interval 0 ≤ *t_s_* <*T* − Δ*t*.

To analytically calculate the main quantities of the simulations, ΔΩ and Δω, is rather convoluted, if even possible. Let *i* be the vertex that is selected at random in the vaccination protocol (called *I* in the main text), and let *j* denote the vertex chosen by the selection criteria of the immunization schemes. In this Supplementary Information, we focus on another quantity, *K_j_*, that is positively correlated with ΔΩ and Δω. *K_j_* denotes the number of neighbors that *j* will be in contact with in the future (clearly a better predictor of *i*’s importance in disease spread from the present time *t* onwards than is the total degree *k_j_*). In epidemic simulations on static networks, the importance of a vertex is proportional to the degree squared—the probability of getting the disease is proportional to the degree, as is the expected number of others to whom the vertex could spread the disease [2]. Since the activity along one edge is independent of the other edges, the possible advantage of *Recent* and *Weight* over *NV* is related to the activity over the edge between the *i* and *j*. Let $\mathbb{E}^{\mathrm{strategy}}$*K*_j_ be the expectation value of *K_j_* after the vaccination, then the independence of edges gives the relative *K_j_* -advantage of the strategy as

$\Delta K_{j}=\frac{\mathbb{E}^{\mathrm{strategy}}K_{j}}{\mathbb{E}^{\mathrm{NV}}K_{j}}=\frac{x_{\mathrm{model}}\left( t \right)\left( k_{j}-1 \right)+P(k_{j},t)}{x_{\mathrm{model}}(t)k_{j}},$ (1)

where *x*_model_(*t*) is the probability that a random edge is active after time *t* for a particular contact model. For the varying activity model, *x*(*t*) is equal to the probability that a random edge has *τ* < *t*, i.e. 1 − *t /T* . For the partner-turnover model, *x*(*t*) is the chance *t_s_* < *t* which is also 1 − *t / T* . We can thus set *x* =1 − *t / T* and drop the model- and *t*-dependence in Eq. 1. In our simulation *t* = 7,500 and *T* = 10,000 giving *x* =1/4. Eq. 1 tells us that a strategy is better than *NV* if $P\left( k_{j},t \right)>x$ and worse if $P\left( k_{j},t \right)<x$, for the rest of this Supplementary Information we will focus on this relation and derive $P(k_{j},t)$ for the various contact models and vaccination schemes.

**The partner turnover model**

*The Recent strategy*

To compute $P(k_{j},t)$ for the *Recent* strategy applied to the partner turnover model we have to consider three cases. These are that all *i’*s contacts have happened already; that none of them have happened; or the one is still going on. The first case happens if *t* < *t_s_* for all *i*’s edges, i.e. with probability $x^{k_{i}}$. If this is the case, there will certainly be more contacts along (*i*,*j*). The second case, that all of *i*’s contacts have already taken place has a probability of ${(1-x)}^{k_{i}}$ in which case no more contacts along (*i*,*j*) will happen. Otherwise, if some of the contacts are in an ongoing relationship around *t*, the probability of another contact is 1. Summing this up, we get

$P\left( k_{j},x \right)=x^{k_{j}}+1-x^{k_{j}}-\left( 1-x \right)^{k_{j}}=1-{(1-x)}^{k_{j}}.$ (2)

Or, replacing the degrees *k_j_* by their averages

$P\left( x \right)=1-{(1-x)}^{2M/N}.$ (3)

Inserting average values for our simulation into Eq. 3 gives *P* = 0.683... . Thus we have *P* > *x*, predicting a better performance of *Recent* than *NV* on the PT contact structure.

*The Weight strategy*

Let *j* be the neighbor of *i* picked by the *Weight* method, then unless all edges have contacts left to happen, the edge with the highest weight will be inactive (i.e., never more have contacts). The probability that one edge will be active in the future is *x*, giving the probability that at least one edge will not be passive in the future

$P\left( k_{j},x \right)=\left[ x\left( 1-\frac{1}{\Delta t} \right) \right]^{k_{j}}.$ (4)

In a mean-field approximation, we get

$P\left( x \right)=\left[ x\left( 1-\frac{1}{\Delta t} \right) \right]^{2M/N}.$ (5)

The last factor 1 − 1 / Δ*t* is a correction for the instance that by chance *t* is within Δ*t* from the end, so that there will be no more contacts along that edge. In our case, on average, *P* = 0.0033 ··· < *x* which predicts a worse performance of *Weight* compared with *NV* and *Recent*.

**Varying activity model**

*The Weight strategy*

To calculate *P* for the *Weight* strategy applied to the varying-activity model, we note that if *τ* > *T* / 2, there will only be one contact along the edge, so the probability of a future contact is *x*. If *T* / 3 < τ ≤ *T* / 2, there will be two contacts along the edge so this interval will contribute with a probability, 1 − 2(1 − *x*), of another contact. In general, if *n* < 1 / (1 − *x*), the interval *T* / (*n* + 1) ≤ *τ* < *T* / *n* will contribute with *n*(1 − *x*) to the probability 1 – *P* that there will not be another contact over the edge. If *n* ≤ 1 / (1 − *x*), the

$1-P\left( x \right)=\sum_{\nu=1}^{n} \left( \frac{1-x}{\nu}-\frac{1}{\nu+1} \right)=\left( 1-x \right)H_{n}-H_{n+1}+1.$ (6)

where *H_n_* is the *n*th harmonic number for *n* chosen such that

$\frac{1}{n+2}\leq x\leq\frac{1}{n+1}$ which gives $n+1\leq\frac{1}{x}\leq n+2$ (7)

*P* will thus be a piece-wise linear function with a steeper slope for larger *T*. Using the logarithmic divergence of harmonic numbers, we can approximate *P* as

$P\left( x \right)\approx ln\left( 1+\frac{1}{n} \right)+x(\gamma+\ln n) ,$ (8)

where γ is the Euler–Mascheroni constant 0.5772 ... . Furthermore, we can approximate 1 / *x* by its the midpoint of the interval (Eq. 7), and get

$n\approx\frac{1}{x}-\frac{3}{2} .$ (9)

In our simulation *x* = 1/4 so $n\approx5/2$. Inserting Eq. 9 into Eq. 8 gives

$P\left( x \right)\approx0.7098$ (10)

So, just as the simulation our calculation predicts, *Weight* does better than *NV* for the varying activity model.

*The NV strategy*

Consider a segment *ν* in the varying activity model. The chance an edge will not happen again is ${v_{\nu}}/{V_{\nu}}$, see Fig. S5 (it is the “window”—the part of the segment not having another slanting line above), where

$V_{\nu}=\frac{1}{\nu}-\frac{1}{\nu+1}=\frac{1}{\nu(\nu+1)}$ (11)

and

$v_{\nu}=\frac{1}{\nu(\nu+1)}-\frac{x}{\nu}$ (12)

giving the probability

$1-x(\nu+1)$ (13)

To get an estimate of *P*(*x*), we have to sum these contributions for all segments where $v_{\nu}>0$. Summing up we get

$P\left( x \right)=1-\sum_{\nu=1}^{\frac{1}{x}-1} [1-x(\nu+1)]$ (14)

where the maximum *ν*-value comes from the constraint $v_{\nu}>0$. For our parameter values we have

$P\left( \frac{1}{4} \right)=1-\frac{1}{2}-\frac{1}{4}=1/4$ (15)

*The Recent strategy*

The calculation for the *Recent* strategy gets more convoluted since one have to link the probability of being most recent with the probability of being under a “window” (where no contact will happen). Large ν-values have fewer (if any) windows, but smaller most-recent windows. We will not derive how these two effects play out—if this is enough to capture *Recent*’s behavior on the Varying Activity model or if one would need to take higher order effects into account.

**Conclusions about contact models**

I these derivation, we derived the relative behavior of our two vaccination strategies on the two contact-pattern models. Instead of the quantities ΔΩ and Δω—our main quantities for evaluating the efficiency of the schemes—we focus on the expected future degree of a vaccinated individual. This quantity, we show analytically, ranks the strategies similarly as concluded in the main article (see a summary in Table S1). Even though the network topology is completely random, so that the effects that the vaccination protocols can utilize are only temporal, there are several aspects of the simulated disease dynamics that our approximation misses—for example cooperative effects where vaccinated vertices that block transmission pathways can contribute to herd immunity. This analytical work can thus not be expected to explain more than a coarse qualitative picture.

**References**

1. Holme P (2005) Network reachability of real-world contact sequences. *Phys Rev E* **71**: 046119.
2. Anderson RM, May RM (1992) *Infectious diseases in humans*. Oxford UK: Oxford University Press.
3. Erdős P, Rényi A (1959) On random graphs I. *Publ Math Debrecen* **6:** 290–297.
4. Moore C, Newman MEJ (2000) Epidemics and percolation in small-world networks. *Phys Rev E* **61:** 5678–5682.

**Table S1. The values of P**. *P* is the probability that the edge between the randomly picked *i* and the vaccinated vertex will not be active in the future. The values assume the model parameters of our simulations. The largest *P*-value gives the largest future expected degree.

|  | *Recent* | *Weight* | *NV* | ranking |
| --- | --- | --- | --- | --- |
| Partner turnover | 0.68 | 0.0033 | 0.25 | *Weight* < *NV* < *Recent* |
| Varying activity | – | 0.7098 | 0.25 | *NV* < *Weight* |
